# EGFR Signaling in Non-Small Cell Lung Cancer: From Molecular Mechanisms to Therapeutic Opportunities

**DOI:** 10.3390/cells11081344

**Published:** 2022-04-14

**Authors:** Silvia La Monica

**Affiliations:** Department of Medicine and Surgery, University of Parma, 43126 Parma, Italy; silvia.lamonica@unipr.it

Lung cancer is the leading cause of cancer death worldwide. The identification of EGFR activating mutations and the development of small molecule EGFR-tyrosine kinase inhibitors (TKIs) have improved the clinical outcome of NSCLC (Non-Small Cell Lung Cancer) patients. Despite the initial efficacy, inevitably patients develop acquired resistance to EGFR-TKIs within a median of 19 months.

The Special Issue entitled “EGFR Signaling in Non-Small-Cell Lung Cancer: From Molecular Mechanisms to Therapeutic Opportunities” focuses on the recent progresses in EGFR-mutated NSCLC translational research highlighting the latest achievements in the identification of new biomarkers and predictors of resistance and in the development of new strategies for overcoming or delaying resistance to EGFR-TKI and. The Special Issue published eight articles consisting of two original articles, eight review articles, and one perspective article.

Recent evidence suggests that miRNA expression profiles could serve as potential biomarkers for predicting patient prognosis and response to targeted therapy and could become a useful tool for monitoring of EGFR-TKI therapy in EGFR-driven NSCLC. In a retrospective study, Leonetti et al. explored the role of specific plasmatic miRNAs (miR-21, miR-27a, and miR-181a) as markers for predicting the EGFR-TKI effects and the risk of developing resistance in EGFR-mutated NSCLC patients. In particular, they collected plasma samples from a cohort of 39 advanced EGFR-mutated NSCLC patients treated with EGFR-TKIs and measured miRNA levels using RT-PCR. Patients who achieved a partial/complete response (PR/CR) had higher basal values of miR-21 compared to those with stability/progression of disease (SD/PD). However, patients who experienced SD had an increase in miR-21, miR-27a, and miR-181a levels compared to patients achieving PR/CR. Moreover, their preclinical studies showed different role of miRNAs expression in the TKI-resistance mechanisms in NSCLC cells harbouring different EGFR mutations. Considering that miR-21 levels are modulated after TKI-treatment, the authors hypothesize that a dynamic evaluation of circulating miRNA levels is better than a single assessment [1].

Recently, there has been a growing interest in liquid biopsies to detect resistance mechanisms to targeted therapies and to monitor the disease evolution given their non-invasive, low-risk, and less expensive nature than tissue biopsies. In particular, the analysis of circulating tumour DNA (ctDNA), which is released by tumour cells into the blood, provides a rapid approach to address the therapeutic response to target therapies and could represent a molecular indicator of PD that can anticipate radiographic and clinical progression. Fernandes et al. reported a prospective study evaluating the therapeutic value of detecting ctDNA mutation in advanced NSCLC patients. They collected 101 plasma samples from advanced NSCLC patients with known oncogenic mutations, mostly EGFR mutations, at different clinically relevant time points (baseline, best response, and progression), and then performed an amplicon-based targeted gene NGS panel. Thus, beyond the initial mutations found in the tumour, they evaluated the disappearance of initial mutations or the appearance of secondary mutations following treatment. Interestingly, in some cases the characterization of oncogenic mutations could be an earlier indicator of PD than radiographic changes. The authors suggest that the targeted NGS of ctDNA could be an important tool to monitor the response of patients with NSCLC to the EGFR-TKI treatment [2].

The review by Karlsen et al. is a comprehensive overview of literature regarding the EGFR target therapy in the NSCLC setting. It begins with a description of the biological function of EGFR and how it is deregulated in NSCLC cases. Moreover, it summarises the currently approved targeted treatment options and describes in detail the complex patterns of resistance, including the intrinsic and acquired resistance mechanisms, such as secondary mutations in the EGFR gene, enhanced signalling in downstream or alternative pathways, histologic transformation, transcriptional regulation, and gene fusions [3].

The review by Suda and Mitsudomi describes the role of the drug-tolerant cells (DTCs) in the acquisition of resistance to EGFR-TKI. The DTCs demonstrate reversible drug insensitivity and survive the early phase of TKI exposure; these cells probably derived from single-cell cloned sensitive cells that do not harbour innate aberrations that confer insensitivity to TKIs. Therefore, DTCs are hypothesized to be an important source of cancer cells that acquire EGFR TKIs-resistance after a long-term exposure to the drug. The authors summarize the features, the potential origin, and the molecular mechanisms that mediate drug tolerance. Understanding the molecular mechanisms of drug tolerance towards EGFR-TKIs could contribute to efforts to develop therapeutic strategies that co-target DTCs [4].

It is well known that although EGFR is an oncogene driver in a subset of NSCLC, the mutation alone is not sufficient to confer a full malignant phenotype. Farnsworth et al. highlight the role of additional genetic and molecular modifications in mediating EGFR mutant tumour progression and in allowing cells to bypass EGFR signalling. In particular, the authors summarize the results of genetic profile screens performed on models of EGFR mutant NSCLC designed to positively or negatively affect sensitivity to EGFR inhibitors. Finally, they suggest a perspective on how these findings could translate into novel combination therapies, targeting both EGFR and its associated dependencies [5].

Belluomini et al. summarize the available data about EGFR-TKIs treatment in both neoadjuvant and adjuvant settings in EGFR mutant NSCLC. They discuss the existing open issue about the opportunity to anticipate the targeted therapy to early stage in EGFR mutant NSCLC. Appropriate endpoint selection for clinical trials, management of disease progression, patients’ selection, and the feasibility to anticipate molecular profiling represent a unique opportunity to change the prognosis of EGFR mutant NSCLC patients [6].

In the context of the metabolic alterations occurring within cancer cells, more attention has recently been given to the reprogramming of lipid metabolism. In particular, the increase of uptake, synthesis, oxidation, and storage of lipids has been demonstrated to have a role in the growth of many types of cancer, including lung cancer. The review by Eltayeb et al. focuses on the role of lipid metabolic reprogramming in lung cancer development and progression and in resistance to therapies, highlighting in particular its connection with EGFR signalling. In addition, the authors summarize the potential therapeutic strategies targeting lipid metabolism for lung cancer treatment, focusing in particular on the combinatorial approaches targeting lipid metabolism with molecular target therapies or with conventional chemotherapy [7].

In a perspective study, Kast et al. built up a proposal to address the problem of the appearance of osimertinib-resistance of cancer cells leading to tumour relapse. The authors developed a five-drug adjuvant regimen (OPALS) designed to enhance the growth inhibition induced by osimertinib and thereby delay the development of resistance. The five OPALS adjuvant drugs are the anti-protozoal drug pyrimethamine, the antihistamine cyproheptadine, the antibiotic azithromycin, the antihistamine loratadine, and the potassium sparing diuretic spironolactone. The authors showed how the OPALS drugs intersecting with NSCLC pathophysiology inhibited its growth, although none of the five adjuvant drugs is currently used individually to treat cancer. Therefore, they argue that co-treatment with OPALS adjuvant drugs could circumvent or delay the development of osimertinib-resistance, suggesting the need for a pilot study of OPALS drugs in combination with current standard treatment [8].

## References

[1] Leonetti A., Capula M., Minari R., Mazzaschi G., Gregori A., El Hassouni B., Papini F., Bordi P., Verzè M., Avan A. (2021). Dynamic evaluation of circulating miRNA profile in EGFR-Mutated NSCLC patients treated with EGFR-TKIs. Cells.

[2] Fernandes M.G.O., Sousa C., Pereira Reis J., Cruz-Martins N., Souto Moura C., Guimarães S., Justino A., Pina M.J., Magalhães A., Queiroga H. (2021). Liquid biopsy for disease monitoring in non-small cell lung cancer: The link between biology and the clinic. Cells.

[3] Karlsen E.-A., Kahler S., Tefay J., Joseph S.R., Simpson F. (2021). Epidermal growth factor receptor expression and resistance patterns to targeted therapy in non-small cell lung cancer: A review. Cells.

[4] Suda K., Mitsudomi T. (2021). Drug tolerance to EGFR tyrosine kinase inhibitors in lung cancers with EGFR mutations. Cells.

[5] Farnsworth D.A., Chen Y.T., de Rappard Yuswack G., Lockwood W.W. (2021). Emerging molecular dependencies of mutant EGFR-driven non-small cell lung cancer. Cells.

[6] Belluomini L., Riva S.T., Simbolo M., Nocini R., Trestini I., Avancini A., Tregnago D., Ferrara M.G., Caldart A., Dodi A. (2021). Anticipating EGFR targeting in early stages of lung cancer: Leave no stone unturned. Cells.

[7] Eltayeb K., La Monica S., Tiseo M., Alfieri R., Fumarola C. (2022). Reprogramming of lipid metabolism in lung cancer: An overview with focus on EGFR-Mutated non-small cell lung cancer. Cells.

[8] Kast R.E., Halatsch M.-E., Rosell R. (2021). OPALS: A new osimertinib adjunctive treatment of lung adenocarcinoma or glioblastoma using five repurposed drugs. Cells.

